# HIF‐1α interacts with Kindlin‐2 and influences breast cancer elasticity: A study based on shear wave elastography imaging

**DOI:** 10.1002/cam4.3130

**Published:** 2020-05-21

**Authors:** Xiaowei Xue, Shaowei Xue, Wenbo Wan, Junlai Li, Huaiyin Shi

**Affiliations:** ^1^ Department of Ultrasound The Second Medical Center of Chinese PLA General Hospital Beijing China; ^2^ Department of Ultrasound The First Medical Center of Chinese PLA General Hospital Beijing China; ^3^ Department of Pathology The First Medical Center of Chinese PLA General Hospital Beijing China

**Keywords:** Breast cancer, E_max_, HIF‐1α, Kindlin‐2, Shear wave elastography

## Abstract

Breast cancer was the most frequent and the second most deadly cancer in women in 2018 in China; thus, early diagnosis of breast cancer is important. Studies have reported that tissue stiffness promotes cancer progression through increased collagen or fibrosis. Shear wave elastography (SWE) is a technique for measuring tissue stiffness. However, the mechanisms underlying cancer tissue stiffness or fibrosis are not entirely clear. Hypoxia‐inducible factor 1 (HIF‐1α) is expressed in response to hypoxia and contributes to tumor progression and metastasis. Kindlin‐2 is an important co‐activator of integrin. We have reported that Kindlin‐2 influences breast cancer stiffness and metastasis. In this study, SWE was used to determine the maximum elasticity (E_max_) of patients before operation or core needle biopsy. The specimens were used for staining. Knockdown, overexpression, co‐immunoprecipitation, and immunofluorescence assays were used to explore the relationship between HIF‐1α and Kindlin‐2. We found that HIF‐1α and Kindlin‐2 were highly expressed in invasive breast cancer and that the expression levels of HIF‐1α and Kindlin‐2 were correlated with E_max_. HIF‐1α interacts with Kindlin‐2. Besides, HIF‐1α and Kindlin‐2 influence the expression of P4HA1, an important protein in collagen biogenesis through the integrin/FAK pathway. Our study first identified a new mechanism of invasive breast cancer stiffness by linking HIF‐1α and Kindlin‐2 to collagen biogenesis. Therefore, based on SWE, E_max_ could be a physical biomarker of invasive breast cancer for early, noninvasive diagnosis, and HIF‐1α and Kindlin‐2 could be pathological markers for early diagnosis and targeted therapy.

## Graphical abstract

The expression levels of HIF‐1α and Kindlin‐2 and E_max_ were correlated with each other. HIF‐1α and Kindlin‐2 influence the progress of collagen biogenesis through integrin/FAK pathway. E_max_ could be a physical biomarker for early, noninvasive diagnosis, and HIF‐1α and Kindlin‐2 could be pathological markers for early diagnosis and targeted therapy.

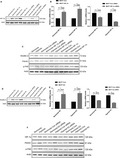

## 1 INTRODUCTION

According to the World Health Organization, breast cancer is the most frequent and the second most deadly cancer in women in China.^1^ Despite the increase in the 5‐year survival rate in breast cancer, early diagnosis, and prognosis of the patients with distant metastasis are unsatisfactory. Shear wave elastography (SWE) is a technique used for detecting tissue by providing quantitative parameters. Breast cancer tissue is stiffer than normal tissue or benign lesions, and the stiffness of cancer facilitates cancer metastasis.^2^, ^3^ However, the mechanisms underlying breast cancer stiffness are not well understood.

Hypoxia‐inducible factor 1 (HIF‐1α) is a transcription factor which plays an essential role in O_2_ homeostasis^4^, ^5^, ^6^, ^7^ and it is composed of HIF‐1α and HIF‐1β subunits.^4^ HIF‐1β is a structural subunit, whereas HIF‐1α is the unique, O_2_‐regulated subunit that determines HIF‐1α activity.^8^, ^9^ HIF‐1α transactivates various genes including heme oxygenase 1, VEGF, IGF‐2, and others.^9^ HIF‐1α is upregulated in multiple human cancers, such as ovarian, prostate, and breast cancers.^10^, ^11^, ^12^

Kindlin‐2 is a member of Kindlin family. It can activate integrins and regulate cell‐matrix adhesion. Kindlin‐2 is dysregulated in many human cancers. Kindlin‐2 was found to be highly expressed in human malignant mesothelioma.^13^ Kindlin‐2 influences breast cancer progression and prognosis^14^ and promotes pancreatic ductal and renal ductal fibrosis.^15^

We initially reported that Kindlin‐2 was highly expressed in breast cancer and its expression level was correlated with breast cancer stiffness and malignancy. However, the interaction between HIF‐1α and Kindlin‐2 and the relevant mechanism of the breast cancer stiffness remains unclear.

In this study, we demonstrated that HIF‐1α and Kindlin‐2 are highly expressed in invasive breast cancer and that both are correlated with its stiffness. Moreover, we found that HIF‐1α interacts with Kindlin‐2 and influences P4HA1, a protein involved in collagen biogenesis, through the integrin/FAK pathway.

## 2 MATERIALS AND METHODS

### 2.1 Patients

The breast tissues used in this study were collected between January 2018 and May 2018. Finally, 66 nodules from 66 patients were included. The ultrasound and SWE detection were performed before surgery or core needle biopsy. Nodules with macrocalcification, pregnancy, lactation, breast implants, ongoing radiation, and chemotherapy, and the presence of scars close to breast lesions were excluded. Histologic analysis confirmed 30 fibroadenomas and 36 invasive breast cancers. The detail information is shown in Table 1. This study was approved by our local Ethics Committee of the Chinese PLA General Hospital. Informed consent was obtained from all patients included in the study. The study was performed in accordance with relevant guidelines and regulations.

**Table 1:** Characteristics of patients and breast nodules

Parameter	Benign	Malignant
Patients (n = 66)	Fibroadenoma (n = 30)	Invasive breast cancer (n = 36)
Age (y)	38.21 ± 9.71	45.35 ± 9.62
Sex	Female	Female
Size（cm）	1.35 ± 0.63	1.79 ± 0.58

### 2.2 Imaging

Breast ultrasonography and SWE were performed by Aixplorer (SuperSonic Imagine,Aix en Provence, France), which contains a sonoelastography unit and a high‐frequency 4‐ to 10.0‐MHz linear‐array probe. SWE parameters (E_max_, E_min_, and E_mean_) were obtained using the same probe. The SWE was performed according to our and other previous studies.^16^

### 2.3 Cell culture, transfection, and treatment

Human breast cell line MCF7 were cultured in DMEM supplemented with 10% fetal bovine serum (FBS) and antibiotics in a 37°C with 5% CO_2_ and 20% O_2_ (normoxia). Hypoxic cells were maintained in a 37°C incubator chamber flushed with a gas mixture containing 5% CO_2_, 94% N_2_, and 1% O_2_ (hypoxia). The transfection assays were performed according to the manufacturer's instruction.

### 2.4 Plasmids and antibodies

Human HIF‐1α, Kindlin‐2 plasmid, HIF‐1α siRNA, and Kindlin‐2 siRNA were generated by HanBio Technology (Shanghai, China). Specific siRNAs targeting human HIF‐1α (HIF‐1α siRNA) and Kindlin‐2 (K2 siRNA) were designed and synthesized by Biomics Biotechnologies Co., Ltd (Nantong, China). HIF‐1α antibody (Affinity, BF0593, USA), anti–Kindlin‐2 antibody (Sigma, K3269, USA), anti–p‐FAK antibody (Abcam, ab4792, UK), and anti–P4HA1 antibody (Proteintech, 12658‐1‐AP, China) were used in this study.

### 2.5 CO‐immunoprecipitation and western blotting

NP40 buffer with protease inhibitors was used to lyse cells for 30 min on ice. For co‐immunoprecipitations, lysates were incubated with targeted antibody (5–10 μg) overnight at 4°C. Then 50 μl of protein A*/*G agarose beads was added and incubated for 2 hours at 4°C. After washing by NP40 buffer for three times, the immunoprecipitated complexes were subjected to western blot with the targeted antibodies. The primary antibodies used for western blot are anti–HIF‐1α human monoclonal antibody (Affinity, BF0593, USA), anti–Kindlin‐2 rabbit polyclonal antibody (Sigma, K3269, USA), anti–p‐FAK rabbit monoclonal antibody (Abcam, ab4792, UK), and anti–P4HA1 rabbit monoclonal antibody (Proteintech, 12658‐1‐AP, China).

### 2.6 Immunofluorescence

For immunofluorescence, cells cultured on glass coverslips were fixed with 4% paraformaldehyde and permeabilized with 0.1% NP40 at room temperature. 5% bovine serum albumin was used to blocked endogenous antigen. Then cells were incubated overnight at 4°C with targeted primary antibody. After washing by PBS three times, cells were incubated with relevant secondary antibody for 1 hour at 4°C. Cells were stained with 4’,6‐diamidino‐2‐phenylindole (DAPI) and mounted. Images were taken photos under a fluorescence microscope (Olympus, Tokyo, Japan).

### 2.7 Immunohistochemical and tissue double immunofluorescence assay

Tissue sections went through deparaffinized, rehydrated, antigen retrieval. Then sections incubated with primary antibody at 4°C overnight. The corresponding second antibodies were applied. After diaminobenzidine and counterstaining with hematoxylin or 4’,6‐diamidino‐2‐phenylindole (DAPI),^17^ the sections were examined by a microscope (Olympus, Tokyo, Japan) and analyzed by using Image Pro Plus 6.0 software.^18^

### 2.8 Real‐Time PCR

Total RNA was isolated from cells using Trizol reagent (Invitrogen) and cDNA was synthesized. Two‐step real‐time polymerase chain reaction (PCR) was performed using the SYBR Green Mix (Roche) and a LightCycler®96 detection system (Roche) according to manufacturer's instructions. The primers for HIF‐1α and Kindlin‐2 were as follows: HIF‐1α^19^ forward primer, 5ʹ‐TGAAGTGTACCCTAACTAGCCGA‐3ʹ, reverse primer, 5ʹ‐GTTCACAAATCAGCACCAAGC‐3ʹ; Kindlin‐2 forward primer, 5ʹ‐TGTCTCCCCGCTATCTAAAAAAGT‐3ʹ, reverse primer, 5ʹ‐TGATGGGCCTCCAAGATTCT‐3ʹ. All mRNAs were normalized to the mRNA level of Actin gene.

### 2.9 Statistical analysis

Student's t test was used to analyze differences between parameters. Correlations between IHC and E_max_ of breast nodules were evaluated with Pearson's test, and coefficients were calculated.

## 3 RESULTS

### 3.1 HIF‐1α and Kindlin‐2 are highly expressed in breast cancer and correlated with breast cancer stiffness

**Figure 1:**
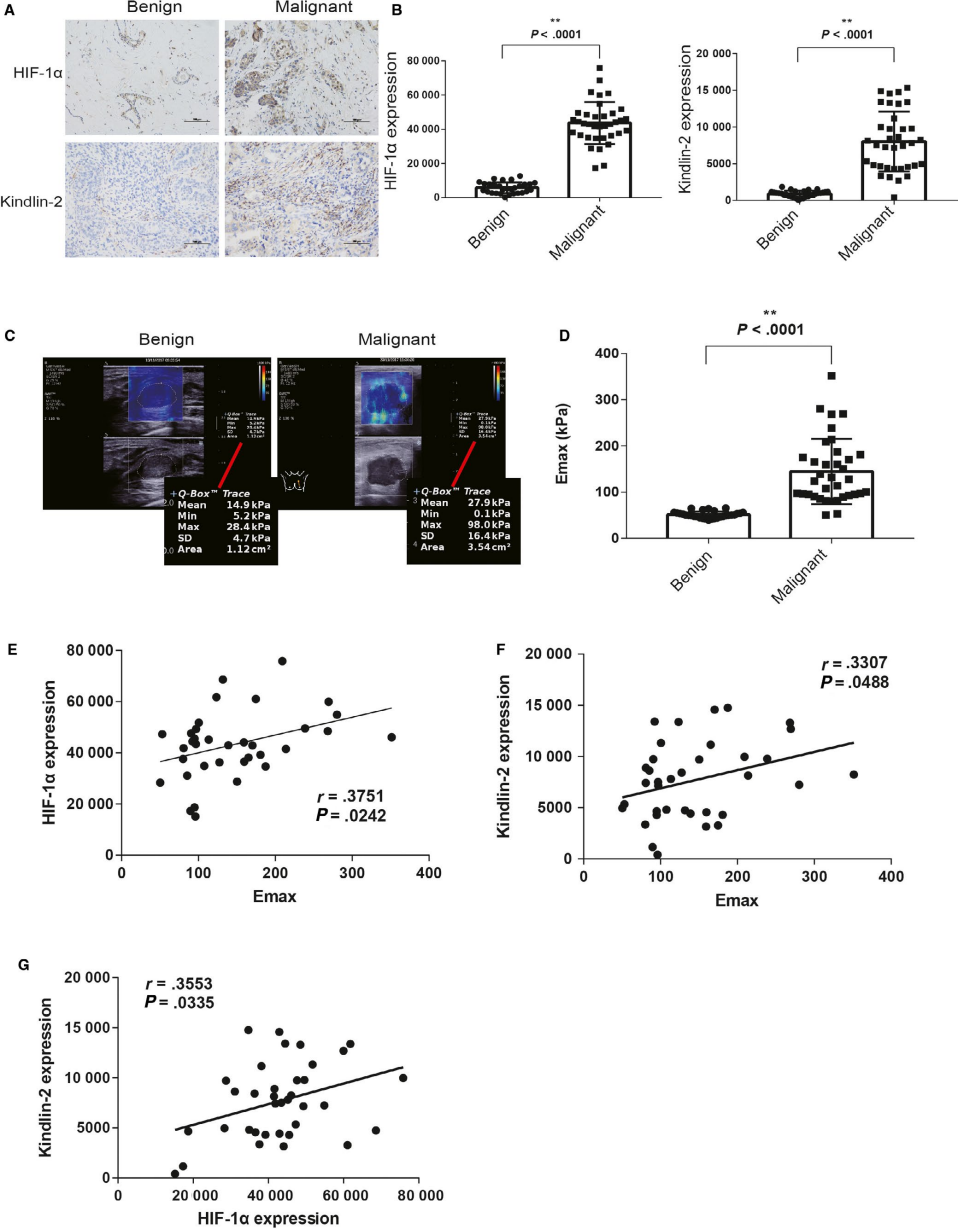
HIF‐1α and Kindlin‐2 are highly expressed in breast cancer and are correlated with breast cancer stiffness. (A) Representative images from immunohistochemical (×200) staining show expression of HIF‐1α and Kindlin‐2 in benign and malignant breast nodules, respectively. (B) Quantitative analysis shows that the average expression levels of HIF‐1α and Kindlin‐2 in breast cancer were significantly higher than those in benign breast nodules. (C) Representative SWE images of benign and malignant breast nodules. (D) The E_max_ of breast cancer is significantly higher than that of benign breast nodules. (E) The correlation between HIF‐1α expression and E_max_ of invasive breast cancer (r = .3751). (F) The correlation between Kindlin‐2 expression and E_max_ of invasive breast cancer (r = .3307). (G) HIF‐1α expression correlated with Kindlin‐2 expression in invasive breast cancer (r = .3553). *P < .05, **P < .01

Hypoxic conditions exist in many solid cancers. Hypoxia induces HIF‐1α, a transcription factor that binds to various target genes and activates them.^9^, ^20^, ^21^ Kindlin‐2 overexpression has been reported in breast cancer.^22^ There are studies that found Kindlin‐2 to be involved in renal fibrosis^23^ and breast cancer stiffness. However, little is known about the expression status of HIF‐1α in breast cancer and the relationship between HIF‐1α, Kindlin‐2, and breast cancer stiffness. To this end, we examined the levels of HIF‐1α and Kindlin‐2 in a cohort of 66 patients including 66 nodules, by immunohistochemistry and detected the stiffness of the breast nodules. We found that both HIF‐1α (5802 ± 580.7 vs 43682 ± 2039, *P* < .001) and Kindlin‐2 (899.6 ± 77.39 vs 8018 ± 679.5, *P* < .001) were highly expressed in breast cancer (Figure 1A,B). The E_max_ (52.02 ± 1.309 vs. 144.6 ± 17.76, *P* < .001) (Figure 1C,D) of breast cancer was higher than that of fibroadenoma (Table 2). The optimal cutoff values of E_max_ for the highest Youden index were 65.23 kPa for predicting invasive breast cancer, which yielded 94.44% sensitivity and 96.67% specificity. The area under the curves for E_max_ was 0.97. Furthermore, HIF‐1α, Kindlin‐2, and E_max_ were intercorrelated with each other in invasive breast cancer (Figure 1E–G). Taken together, HIF‐1α and Kindlin‐2 are highly expressed and correlate with invasive breast cancer stiffness.

**Table 2:** Expression of HIF‐1α and Kindlin‐2 and E_max_ of benign and malignant breast nodules

	Benign	Malignant	*P* Value
E_max_ (kPa)	52.02 ± 1.309	144.6 ± 17.76	** *P* < .0001
HIF‐1α	5802 ± 580.7	43 682 ± 2039	** *P* < .0001
Kindlin‐2	899.6 ± 77.39	8018 ± 679.5	** *P* < .0001

Note

There were significant differences between benign and malignant breast nodules.

** *P* < .01.

### 3.2 HIF‐1α and Kindlin‐2 are upregulated and interact with one another in hypoxic conditions in breast cancer

**Figure 2:**
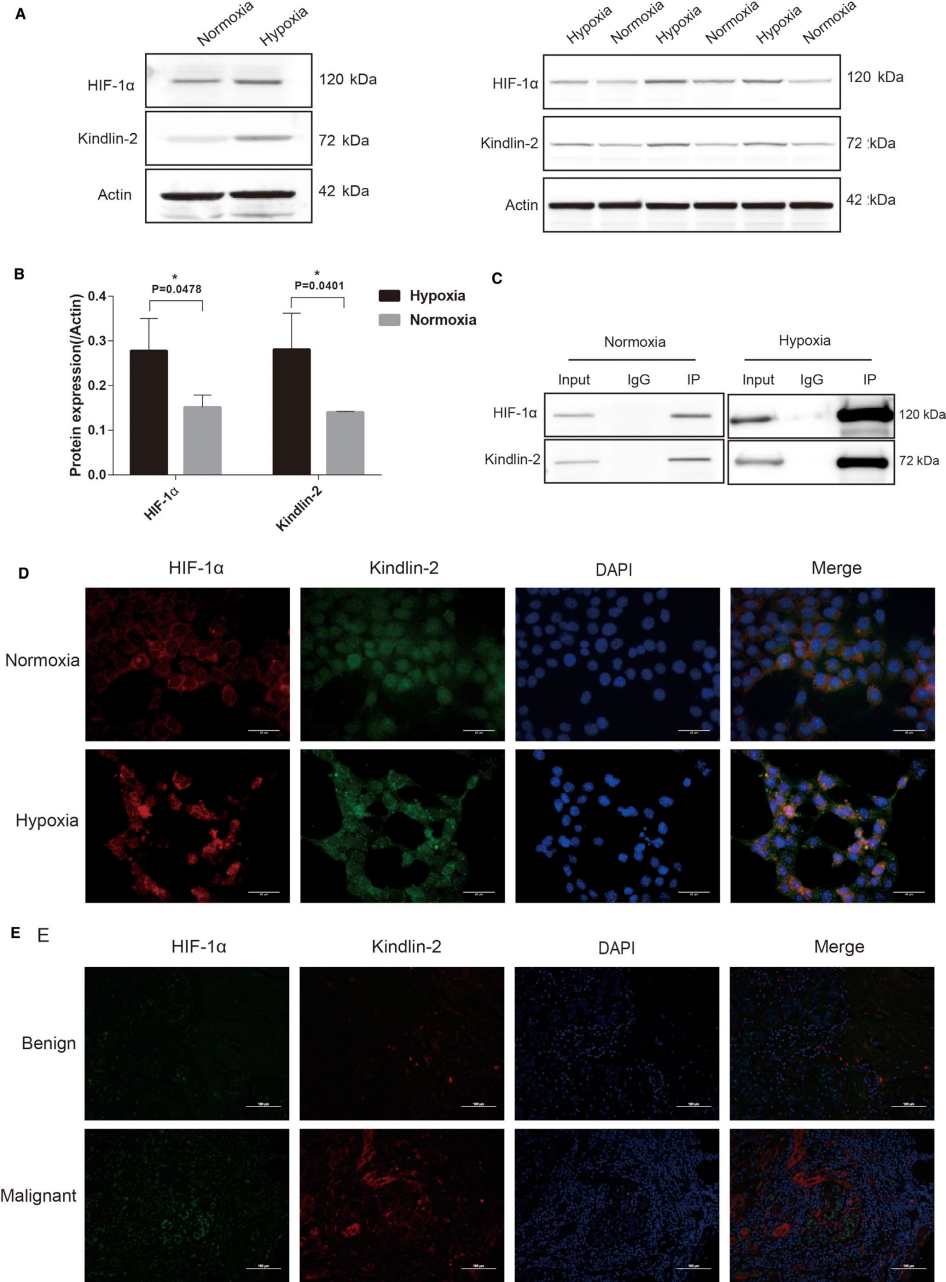
HIF‐1α and Kindlin‐2 are upregulated and interacts with one another in hypoxic conditions. (A) Western blot of extracts of MCF7 cells showing that HIF‐1α and Kindlin‐2 are upregulated in hypoxia. (B) Relative protein levels of HIF‐1α and Kindlin‐2 are higher in hypoxia than in normoxia. (C) Co‐immunoprecipitation assay was performed using lysates from MCF‐7 cells in normoxia and hypoxia with anti–HIF‐1α antibody, followed by immunoblotting with indicated antibodies; the results show that HIF‐1α interacts with Kindlin‐2. (D) Co‐localization of endogenous HIF‐1α with Kindlin‐2. Endogenous HIF‐1α (red) and Kindlin‐2 (green) are stained with specific Abs in normoxia or hypoxia. Nuclei are stained with DAPI (blue) and subsequently visualized by microscopy. Scale bars: 40 μm. (E) Tissue double immunofluorescence assay performed with anti–HIF‐1α (green) and anti–Kindlin‐2 (red) in benign and malignant breast nodules. Nuclei are stained with DAPI (blue) and subsequently visualized by microscopy. Scale bars: 100 μm. *P < .05, **P < .01

Given that HIF‐1α and Kindlin‐2 are overexpressed in breast cancer, we aimed to determine the roles of HIF‐1α and Kindlin‐2 in breast cancer; we detected the expression levels of HIF‐1α and Kindlin‐2 in MCF7 cells under hypoxic conditions. We found that HIF‐1α as well as Kindlin‐2 were upregulated in hypoxia (Figure 2A,B). Moreover, the co‐immunoprecipitation assay indicated that HIF‐1α interacted with Kindlin‐2 (Figure 2C). Furthermore, endogenous HIF‐1α and Kindlin‐2 were highly expressed and co‐localized in cells under hypoxic conditions (Figure 2D). A tissue double immunofluorescence assay found that HIF‐1α and Kindlin‐2 were overexpressed in breast cancer and that they were mainly localized in the nucleus and cytoplasm (Figure 2E). Taken together, these findings demonstrated that HIF‐1α and Kindlin‐2 are upregulated and interact with one another in breast cancer.

### 3.3 HIF‐1α interacts with kindlin‐2 and influences collagen biogenesis by targeting P4HA1 and FAK

**Figure 3:**
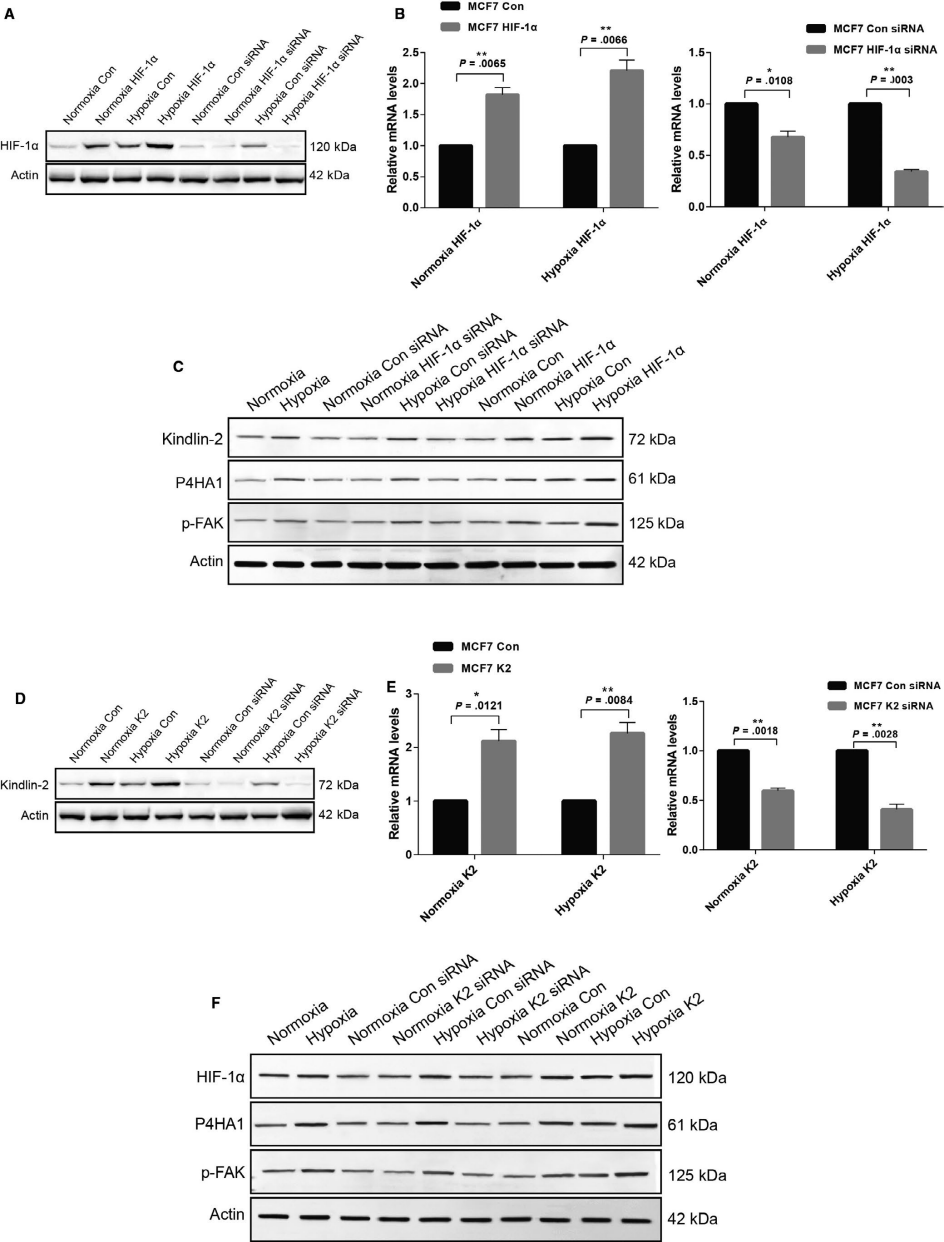
HIF‐1α interacts with Kindlin‐2 and influences collagen biogenesis by targeting P4HA1 and FAK. (A‐C) HIF‐1α was overexpressed with HIF‐1α plasmid or knocked down with HIF‐1α siRNA (A), HIF‐1α mRNA expression level was analyzed by real‐time PCR (B), cell lysates were analyzed by western blot with the indicated antibodies. The expression levels of Kindlin‐2, p‐FAK, and P4HA1 were consistent with HIF‐1α expression (C). (D‐F) Kindlin‐2 was overexpressed with Kindlin‐2 plasmid or knocked down with Kindlin‐2 siRNA (D), Kindlin‐2 mRNA expression level was analyzed by real‐time PCR (E), and cell lysates were analyzed by Western blot with the indicated antibodies. The expression levels of HIF‐1α, p‐FAK, and P4HA1 were consistent with Kindlin‐2 expression (F)

To further confirm the relationship between HIF‐1α and Kindlin‐2, we first adopted gain‐of‐function and loss‐of‐function approaches to specifically overexpress and knockdown HIF‐1α with HIF‐1α plasmid and siRNA (Figure 3A‐B). We found that Kindlin‐2, the co‐activator of integrin was downregulated or upregulated by HIF‐1α (Figure 3C). Next, we adopted Kindlin‐2 gain‐of‐function and loss‐of‐function with Kindlin‐2 plasmid and siRNA (Figure 3D–E) and found that the expression level of HIF‐1α was consistent with that of Kindlin‐2 (Figure 3F). These results further indicated the interaction of HIF‐1α with Kindlin‐2. In addition, the expression levels of phosphorylated focal adhesion kinase (p‐FAK), a main factor of the integrin pathway, and P4HA1, an important protein in collagen biogenesis, were associated with HIF‐1α or Kindlin‐2 expression (Figure 3C,F). It is already known that Kindlin‐2 is an activator of the integrin pathway and that it influences breast cancer stiffness. These results indicated that HIF‐1α interacts with Kindlin‐2 and influences collagen biogenesis by targeting P4HA1 and FAK.

## 4 DISCUSSION

Shear wave elastography is a new ultrasound diagnosis technology. It uses shear wave of the tissue induced by acoustic radiation pulse to visualize and quantify the stiffness of tissue in a real‐time, reliable, and reproducible manner.^24^ Tissue stiffness has become an important parameter in diagnosing potential malignancies or other diseases.^25^, ^26^, ^27^ This is due to the fast metabolism of cancer cells and the complex microenvironment which includes many factors, such as hypoxia and collagen. Studies report that cancer tissues have more collagen fiber deposition and promote cancer stiffness and metastasis.^25^, ^27^ There are many factors promoting cancer stiffness. We have found that Kindlin‐2 and collagen were overexpressed in breast cancer and correlated with breast cancer stiffness. However, there were no reports concerning the relationship between HIF‐1α, Kindlin‐2, and breast cancer stiffness. Hypoxia exists in many solid cancers^28^, ^29^ and its effects are mediated by HIF‐1α.^30^ In tumors, the hypoxic conditions lead to the stabilization of HIF‐1α and to an increased interaction with its co‐activators. In this study, we verified that the expression of HIF‐1α in invasive breast cancer is higher than in fibroadenoma, which is consistent with previous studies.^31^, ^32^

HIF‐1α is overexpressed in many cancers^11^, ^31^, ^33^ and regulates cancer progression through various molecular pathways. In prostate cancer, the inhibition of HIF‐1α by agents that target the PI3K/PTEN/AKT/FRAP pathways contribute to therapeutic efficacy.^34^ In non–small cell lung cancer, the inhibition of HIF‐1α enhances the antitumor effect of radiation through the Notch pathway.^35^ In breast cancer, HIF‐1α activates collagen hydroxylases, which are important for collagen deposition.^36^ Collagen biogenesis requires collagen prolyl 4‐hydroxylases (P4Hs) as the catalyzers and biogenesis. There are three subunits of P4Hs: P4HA1, P4HA2, and P4HA3. P4HA1 is the most common and the best studied one. It was reported that P4HA1 is dysregulated in many cancers; for example, it promotes breast cancer drug resistance,^37^ and plays an important role in the differentiation of glioma stem cells.^38^ However, the underlying mechanism of HIF‐1α in breast cancer stiffness remains unknown. We found that the expression level of HIF‐1α was correlated with breast mass elasticity. Western blotting showed that HIF‐1α and Kindlin‐2 were both upregulated in breast cancer. Using co‐immunoprecipitation, we found that HIF‐1α and Kindlin‐2 interacted with one another. The knockdown and overexpression assays further verified the interaction between HIF‐1α and Kindlin‐2. In addition, the expression levels of p‐FAK (in the integrin pathway) and P4HA1 (in collagen biogenesis) were downregulated or upregulated after HIF‐1α or Kindlin‐2 knockdown or overexpression. It was reported that Kindlin‐2 is an integrin activator and is involved in cell adhesion through the integrin pathway.^36^, ^39^ Collectively, HIF‐1α influences collagen biogenesis (tissue stiffness) through the integrin/FAK pathway by targeting Kindlin‐2.

In summary, we found that HIF‐1α promotes breast cancer stiffness through the integrin/FAK pathway by interacting with Kindlin‐2. HIF‐1α, Kindlin‐2, and E_max_ could form a new panel of diagnostic markers and therapeutic targets for breast cancer. However, the generic role of HIF‐1α in vivo should be investigated in the future using mice models. It will provide a better understanding of the biological interactions between HIF‐1α, Kindlin‐2, and breast cancer stiffness.

## CONFLICT OF INTEREST

The authors declare no competing financial interests.

## AUTHOR CONTRIBUTIONS

XWX, SWX, JLL, and HYS conceived and designed the experiments. XWX and SWX performed the experiments. JLL, HYS, WBW, and SWX participated in clinical sample and clinical data collection. XWX analyzed the data. XWX, SWX, and JLL wrote the main manuscript text. All authors read and approved the final manuscript.

## Data Availability

The data used and/or analyzed during the current study are available from the corresponding author on reasonable request.
